# What Are the Predictors of Self-Reported Change in Physical Activity in Older Adults with Knee or Hip Osteoarthritis?

**DOI:** 10.1007/s12529-022-10080-y

**Published:** 2022-03-23

**Authors:** Erwin C. P. M. Tak, Lisanne M. Verweij, Astrid M. J. Chorus, Marijke Hopman-Rock

**Affiliations:** 1 Tak Advies en Onderzoek, Voorschoten, the Netherlands; 2 Department of Quality and Patient Care, Erasmus MC, University Medical Centre, Rotterdam, the Netherlands; 3 National Health Care Institute, Diemen, the Netherlands; 4 Department of Public and Occupational Health, AmsterdamUMC (Location Vumc), Van der Boechorststraat 7 1081BT, Amsterdam, the Netherlands

**Keywords:** Aging, Behavior, Pain, Exercise, Coping

## Abstract

**Background:**

Although physical activity (PA) has been shown to be beneficial in older adults with osteoarthritis (OA), most show low levels of PA. This study evaluated if self-efficacy, attitude, social norm, and coping styles predicted change in PA in older adults with OA in the knee and/or hip.

**Methods:**

Prospective study following 105 participants in a self-management intervention with baseline, post-test (6 weeks), and follow-up (6 months). Univariate associations and multivariate regression with self-reported change in PA as the dependent variable were measured. Potential predictors in the model: demographic, illness-related, and behavioral variables (attitude, self-efficacy, social norm, and intention), coping style, and pain coping.

**Results:**

Forty-eight percent of participants reported increased PA at 6 weeks and 37% at 6 months which corresponded with registered PA levels. At 6 weeks, use of the pain coping style “resting,” intention, and participation in the intervention was univariately and multivariately, positively associated with more self-reported change, whereas being single and less use of the pain coping style “distraction” predicted less change. Higher pain severity only predicted less change multivariately. At 6 months, univariate associations for age, general coping style “seeking support,” and participation in the intervention were found; higher age was associated multivariately with less self-reported change.

**Conclusion:**

At short term, self-reported change of PA was predicted by the behavioral factors intention and several pain coping styles. Together with other predictors of self-reported change (pain severity, higher age, being single), these could be addressed in future interventions for enhancing PA in older adults with OA.

**Supplementary Information:**

The online version contains supplementary material available at 10.1007/s12529-022-10080-y.

## Introduction

Osteoarthritis (OA) is the most common form of arthritis, with knee and hip joints affected most. OA leads to pain, tenderness, swelling, and decreased function of weight-bearing joints which in turn could lead to disability [1].

As there is no direct cure for OA, the prevention and management of its consequences are important. One of the most widely known and recommended non-pharmacological methods for the effective management of OA is physical activity (PA) [2]. Any movement of your body requiring energy is considered physical activity. Exercise (a subcategory of PA as it is usually structured and planned PA) helps regain muscular condition, balance, and joint stability [3] which improves physical functioning and general well-being and reduces pain [4]. Regular daily PA has preventive and therapeutic effects [5], while avoidance of activity predicts an increase of limitations in patients with knee OA [6]. Walking has also been found to be equally effective in managing knee OA as has home-based quadriceps exercise training [7]. In knee OA, obesity is one of the modifiable risk factors that also can be targeted by PA, preferably combined with dietary interventions [8].

Despite these positive findings, older adults with OA show low levels of maintaining exercise and low compliance with PA guidelines [9]. Greater adherence to recommended exercise programs predicts long-term benefits [7, 10]. More insight into which factors determine PA behavior in older adults with OA can contribute to more effective intervention strategies to increase quality of life and prevent dependency in older adults [11]. Several studies have found that socio-demographic (e.g., lower age, male gender, and higher education), health (e.g., normal weight, no physical limitations, pain, or fatigue), and healthcare factors (i.e., having been advised by a health professional that PA benefits osteoarthritis) are associated with increased levels of PA [12–14].

Individual behavioral aspects such as self-efficacy (an individual’s belief in his or her capacity to execute behaviors), attitude, and pain-coping strategies are all considered possible determinants of PA behavior and thereby on the impact of OA on physical performance and disability [15–19].

More insight into how these factors interact with each other and predict *actual* PA behavioral change in older adults with chronic conditions is needed. The answer still remains a gap in knowledge and could be of importance for designing future more effective interventions. A longer version of this manuscript has been published in the PhD dissertation of the first author [20].

## Methods

### Study Design

Data of 105 participants of an earlier randomized controlled trial (RCT) of a self-management program for patients with OA of the knee and or hip were used [21]. Potential participants were recruited through advertisements in a local newspaper. Inclusion criteria were OA of the hip and/or knee diagnosed by radiographic and clinical criteria, age between 55 and 75 years, and not being on a waiting list for joint replacement.

The intervention program consisted of 6 weekly sessions of 2 h health education by a peer and physical exercises taught by a physical therapist (for details, see Textbox 1). Data were collected by questionnaires, interviews, and physical examination at baseline, at 6 weeks, and at 6-months follow-up. A more detailed study design is described elsewhere [21]. The study protocol was approved by the TNO Medical Ethics Committee. All the participants gave informed consent to the study and human rights were respected.

In order to study factors that determine PA behavior, participants from the earlier RCT were analyzed as one group, controlling for participation in the intervention.

Textbox 1 Description of the intervention [21] Description of the Intervention

The program consisted of 6 weekly sessions of 2 hours. Maximally 15 participants took part in each group. The first hour of each session was guided by a peer educator and the following topics were discussed: pathophysiology of OA, lifestyle and physical activity, pain management, the importance of weight reduction and diets, ergonomic aspects, and medical aspects of OA (treatments, radiographs). Additionally, questions were answered by an invited occupational therapist and a general practitioner (GP). The course included the use of a pain diary and personal goal planning, and interactive methods in the group. Peer education has a known empowerment effect^1^.

In the second hour the participants were taught an exercise program by a physical therapist. Fifteen minutes of each session were spent on education about the balance between rest and activity, preferable types of activity, and how to incorporate them in a daily lifestyle, and practical advice on physical activity, such as the benefits of walking. Participants learned the exercises of the program, which consisted of warming up exercises, exercises for the knee and hip (independently of the site of major pain), and a cooling down including relaxation exercises. All exercises were performed with the help of a chair, and alternatives were offered to participants who preferred to remain seated. Dynamic exercises were alternated with static exercises and a standard resistance protocol was used.

All educational information, addresses of relevant organizations, and the whole exercise program were written up in a course book for the participants. Participants were encouraged to do the exercises at home at least 3 times a week.

### Data Collection

To measure changes in PA behavior (the outcome variable), participants were asked: “Has OA caused you to exercise^2^ more or less over the past 6 weeks/6 months?” Answers were given on a 5-point Likert scale ranging from “much more” to “much less” PA behavior and was dichotomized into more PA at 6 weeks versus the same amount or less PA (model 1), and more PA in the past 6 months versus the same level or less PA (model 2). In two participants, answers on PA behavior outcomes were missing, leaving 103 participants included in the analysis.

To additionally assess PA levels for comparison, the Voorrips questionnaire was conducted by an interviewer at baseline and at 6 months [22]. The reliability (0.89) and validity (0.78) of the questionnaire are good. Respondents were asked to report habitual physical activities over the past (half) year. Questions cover three areas: household activities (mean score of 10 items ranging from “very active” to “inactive”), sport activities (type of activity, intensity, hours per week, and months per year, for a maximum of two activities), and leisure time activities (type of activity, intensity, hours per week and months per year, for six activities maximum). Sport and leisure activity scores were calculated by an equation multiplying the intensity, hours per week and period of the year. The PA levels are reported with mean and SD of continuous data. If tertiles were used this resulted in a total PA score according to which participants were finally classified as having a high, medium, or low level of PA.

The baseline variables age, sex, marital status, education, income, and work status (paid or voluntary work) were identified as demographic variables. There were five categories of marital status: married living together, not married living together, divorced, widowed, and single. Education was divided into three categories following the Dutch educational system: primary education (0–8 years), secondary education (9–16 years), and college/university (17 years and older). Income was classified into categories low, middle, and high income levels in euros (< €908; €908–€1360; > €1360). All participants were white European people.

Duration of joint complaints was assessed by asking “how many years ago did your first OA complaints arise?” Answer categories were < 1 year, 1–3 years, 3–10 years, 10–20 years,” and > 20 years ago. Self-reported joint complaints (right or left hip, right or left knee) were noted by participants if present.

Body mass index (BMI) was calculated as length and weight expressed as kilograms per square meter. Participants were classified as normal weight (BMI < 25), overweight (BMI 25–30), and obese (BMI ≥ 30). Comorbidity, including use of medication, was assessed during the interview by reading out a list of 25 chronic conditions to the participants and asking them if they had any of these disorders and, if so, what sort of medication they took for it. Pain severity, pain tolerance, and fatigue over the previous month were measured on a 10-cm VAS scale ranging from 0 to 100. A higher score indicates more pain/fatigue. Disability was evaluated using the Sickness Impact Profile (SIP) subscale physical functioning which comprises self-reported statements on ability to carry out activities in the area of household management, body care, and movement and mobility. Scores are summarized and presented as a percentage of maximum dysfunction, ranging from 0 to 100%. The higher the score, the higher the level of disability [23].

Behavioral variables were based on the theoretical model of attitude, social norm, and self-efficacy (ASE) of de Vries [24]. The ASE model illustrates how factors contribute to intention to display a desired behavior. Attitude comprised two constructs: the expectations of consequences of certain behavior (e.g., beliefs) and the value given to those expectations (e.g., evaluations). Regarding beliefs, the participant was given a list of six activities and asked “how much benefit do you think the following activities will have on your functioning in general?” The answering scales were “a lot of benefit,” “a little benefit,” and “no benefit.” The same six questions were asked on evaluations; “how important do you feel these benefits are to the following activities” with answering scales “important,” “a little bit important,” and “not important.” Where one item was missing, participants (*n* = 21) were given the statistical mean value. If more than one item was missing per construct, cases were excluded from analysis (*n* = 19). A sum score of attitude to PA was calculated by multiplying the beliefs and evaluations of each question per respondent, followed by adding up the scores of the six questions [25]. A higher score indicated a more positive attitude.

Social norm was operationalized as the perceived opinion of important others (e.g., normative beliefs) and the personal value given to these opinions (e.g., motivation to comply). Normative beliefs were assessed by asking “how do you think your near environment would react if you were to undertake more PA?” with answers “positive, negative or neutral.” The motivation to comply was measured by asking “how important is the opinion of your near environment” on a 5-point answer scale “very important” to “not important.” A social norm score was calculated by multiplying the normative beliefs of each participant with their motivation to comply [25]. A higher score indicates a more positive social norm.

Self-efficacy is one’s belief in being able to carry out a certain behavior. Self-efficacy was measured by asking “do you believe you will succeed in exercising more?” on a 10-cm VAS scale, with a higher score indicating “no, I will not succeed.” This scale was developed by Lorig and Holman [26] to measure perceived self-efficacy in patients with rheumatic disease.

Finally, intention was measured by asking “do you intend to engage in more PA?” Answers “definitely yes,” “probably yes,” “probably no,” and “definitely no” were dichotomized into 1 = yes and 0 = no.

Coping styles in general were assessed at baseline by the short version of the Utrecht Coping List (UCL) which views coping as a personality trait and measures how people deal with health and illness in general. Seventeen items were evaluated by respondents by reporting the frequency of responding to a given problem on a four-point scale from “never” to “very often.” Items were divided into four categories: active problem solving (5 items, such as “seeking ways to solve the problem”), seeking support (5 items, e.g., “asking someone for help”), avoidance (5 items, e.g., “doing other things to avoid thinking about the problem”), and a religious response (2 items, “praying” and “thinking the situation is inevitable because it comes from a higher power”). A higher score indicated more use of the coping style. The short version of the UCL has been found to be valid and reliable [27].

The Pain Coping Inventory (PCI) is a Dutch pain-specific coping instrument developed by Kraaimaat and van Schevikhoven [28]. The PCI assesses how people deal with pain. A four-point answering scale was used to identify how often a certain behavior is carried out (“never” to “very often”). The 35 items were categorized into six pain coping scales: worrying about pain (“I think the pain will get worse”), distraction by engaging in pleasant activities (e.g., “I seek distraction by diverting my attention to reading, music, watching television, or something similar”), resting (e.g., “I restrict myself to simple activities”), pain transformation (e.g., “I imagine the pain to be less severe than it really is”), retreating (e.g., “I ensure I will not be disturbed by intrusive noise”), and reducing demands (e.g., “I make sure I do not get anxious”). A higher score indicated more use of a pain coping style.

Participation in the intervention program and baseline PA (continuous score as calculated by the Voorrips questionnaire) were included as confounders.

### Analysis

Descriptive statistics of all participants on baseline characteristics were calculated. Results for self-reported change in PA behavior and PA levels at 6 months are compared to see if participants’ reports correspond.

To study which factors predicted self-reported change in PA behavior at 6 weeks and at 6 months, univariate comparisons were made between possible predictors and the two models using chi-square tests for categorical variables and *t* tests for continuous variables. All participants were categorized as either carrying out less or the same PA or more PA. In the first step of the (explorative) analysis, all study variables were entered. These variables have been chosen based on the short literature review described in the introduction. Univariate associations of *p* < 0.20 (see Table 3 in the “Results” section) were required for entry into the multivariate model with the exception of pain severity which was added to the multivariate model due to its great impact on patients suffering from OA [29, 30].

**Fig. 1:**
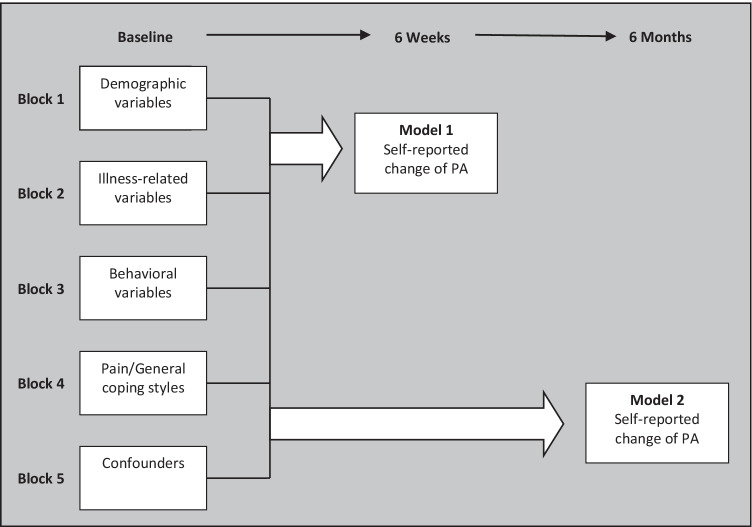
Blocks of independent variables, time of measurement, and two models of change in physical activity (PA) behavior used in multivariate stepwise backward logistic regression analyses

In the second step of the analysis, multivariate stepwise backward logistic regression analyses were first conducted between independent predictors and more PA behavior at 6 weeks (model 1) and at 6 months (model 2). Predictors were tested in five blocks of variables (Fig. 1); demographic variables were tested in block 1, followed by illness-related variables in block 2, behavior variables in block 3, pain coping styles and coping styles in general in block 4, and confounders in block 5. Confounders were entered into the last step of the model to reduce its effects on other variables. Intention was entered as an extra step after attitude to prevent obscuring the multivariate model, as the ASE model shows that attitude influences intention.

In analyzing the two models, changes in *ß*, *p* values, and Nagelkerke *R*^2^ were calculated. Hosmer and Lemeshow’s goodness-of-fit tests were conducted to assess if non-significant chi-square goodness-of-fit value was present. Correlations between predictors were assessed to test for multicollinearity. Correlations higher than 0.50 were excluded to ensure stable regression analyses. Finally, missing analyses were conducted using chi-square tests and *t* tests to assess if excluded participants were similar to valid participants on demographic variables age, sex, marital status, education, income, and illness-related variable body mass index.

To check whether the results for self-reported change could be replicated for PA levels as measured with the Voorrips questionnaire at 6 months, possible predictors of these were tested with the same procedure. *P* values less than 0.05 were considered statistically significant. Statistical analyses were performed using SPSS.

## Results

Baseline characteristics of the total study population (*n* = 105; Table 1) showed that most participants were women, married and living together, and overweight or obese and had completed secondary or higher education. Around one-third were engaged in paid work or voluntary work. Of participants reporting other chronic diseases, OA of the hands, severe back complaints, and high blood pressure were most frequently reported. Almost all participants reported complaints in knee and/or hip joints, with 30% reporting complaints in three or four joints. Knee complaints were twice more prevalent than hip complaints. Radiological OA (ROA) of the knee was two to three times more prevalent than hip ROA. Disability in the domain of physical functioning was low, with participants indicating only 6% maximum dysfunction.

**Table 1:** Baseline characteristics (*n* = 105)

**Demographic variables**	
Sex, % female	82.9
Age, years, mean (SD)	65.5 (5.5)
Marital status, %	
Married, living together	66.7
Not married, living together	2.9
Divorced	7.8
Widowed	7.8
Single	11.8
Education, %	
Primary	22.2
Secondary	52.5
College/university	25.3
Income, %	
< €908	39.5
€908 to €1360	34.9
> €1360	25.6
% paid work	14.0
% voluntary work	34.7

**Illness-related variables**	
BMI, mean (SD)	27.5 (4.2)
% normal weight	29.1
% overweight	48.5
% obese	22.3
Number of diseases, mean (SD)	2.5 (1.6)
% prescribed medicine	45.5
% non-prescribed medicine	29.4
Fatigue past month^a^, mean (SD)	40.4 (22.1)
PA-levels (Voorrips), mean (SD)	11.8 (7.2)
Disability (SIP-subscale physical functioning), mean (SD)	5.8 (7.0)

**OA–related variables**	
Duration of complaints, %	
< 1 year ago	3.1
1 to 3 years ago	22.7
3 to 10 years ago	36.1
10–20 years ago	19.6
> 20 years ago	18.6
Joints with complaints, %	
0 joints	2.0
1 joint	23.5
2 joints	45.1
3 joints	18.6
4 joints	10.8
Pain severity past month^b^, mean (SD)	43.3 (24.4)
Pain tolerance past month^c^, mean (SD)	31.4 (21.8)
Morning stiffness, %	11.4
Joints with self-reported complaints, %	
Hip, left	34.3
Hip, right	25.5
Knee, left	63.7
Knee, right	67.6
Joints with ROA, %	
Hip, left	21.6
Hip, right	15.4
Knee, left	46.6
Knee, right	46.6

**Behavior variables and coping (mean, SD)**	
Attitude	37.9 (11.1)
Social norm	7.3 (4.1)
Self-efficacy	41.9 (24.5)
Intention, % yes	56
Active problem solving (UCL)	2.3 (0.7)
Seeking support (UCL)	1.7 (0.5)
Avoidance (UCL)	2.2 (0.4)
Reacting religious (UCL)	1.5 (0.7)
Pain transformation (PCI)	2.1 (0.6)
Distraction (PCI)	2.4 (0.6)
Reducing demands (PCI)	2.3 (0.6)
Retreating (PCI)	1.6 (0.5)
Worrying about pain (PCI)	1.7 (0.5)
Resting (PCI)	2.3 (0.6)

*BMI* body mass index, *OA* osteoarthritis, *ROA* radiological osteoarthritis, *UCL* Utrecht Coping List, *PCI* pain coping list

^a^Measured on a VAS scale, 0 = not tired

^b^Measured on a VAS scale, 0 = not severe

^c^Measured on a VAS scale, 0 = tolerable

Around 48% of respondents reported they had become more physically active after 6 weeks compared with baseline, 45% remained stable, and 8% reported less PA (Table 2). At follow-up, 6 months later, 38% reported that they had increased their PA behavior on comparison with baseline. A large proportion of all participants who reported more PA at 6 weeks also reported more PA at 6 months (*n* = 28, 58%) or maintained the level (*n* = 19, 40%). A few participants were doing less at 6 months than at 6 weeks (*n* = 11). To assess the correspondence between self-reported change in PA behavior and self-reported PA levels using the Voorrips questionnaire, Table 2 shows the mean change in PA level per change group at 6 months. Although only the “more” group reported a rise in PA levels, compared to a minor decline for the “same” and “less” group, there was no statistically significant difference in mean change between the three groups (*F* = 2.6; *p* = 0.078).

**Table 2:** Number of participants who reported change in physical activity behavior at 6 weeks and 6 months (*n* = 101) and corresponding changes in self-reported physical activity levels (Voorrips (22))

**Physical activity behavior at 6 weeks**	**Physical activity behavior at 6 months**			
	**More**	**Same**	**Less**	**Total**
**More**	28	19	1	48
**Same**	7	31	7	45
**Less**	3	2	3	8
**Total**	38	52	11	101
**Self-reported physical activity levels (Voorrips), mean (SD)**				
Change from baseline	3.4 (9.5)	−0.6 (7.0)	−0.7 (12.7)	0.9 (9.3)

Univariate associations between baseline characteristics and PA behavior are presented in Table 3.

**Table 3:** Univariate chi-square and *t* tests of predictors with three groups of change in physical activity behavior at 6 weeks and 6 months (*n* = 103)

	**Physical activity behavior** **—at 6 weeks**		**Physical activity behavior** **—at 6 months**	
**Baseline characteristics**	**Less and same**	**More**	**Less and same**	**More**
Number	53	50 (48%)	65	38 (37%)
**Demographic variables**				
Sex, % female	87	78	88	76°
Age, years, mean (SD) Min–max	65.4 (5.5) 54–75	65.3 (5.6) 54–75	66.3 (5.2) 55–75	63.3** (5.2) 54–74
Marital status, %^a^				
Married living together	71	63*	68	68
Not married living together	2	4	2	3
Divorced	2	14	5	14
Widowed	6	10	8	5
Single	16	6	16	5
Education, %				
Primary	24	19	20	24
Secondary	50	55	55	51
College/university	26	26	25	24
Income, %^b^				
< €908	35	44	43	33°
€908 to €1360	33	21	31	22
> €1360	33	35	26	44
% paid work	16	12	10	22°
% voluntary work	39	30	28	44°
**Illness-related variables**				
BMI, mean (SD)^b^	27.0 (3.7)	28.1 (4.7)°	26.9 (4.3)	28.5 (3.8)°
% normal weight	31	27	37	16^a^
% overweight	52	45	46	55
% obese	17	29	18	29
Number of diseases, mean (SD)	2.3 (1.7)	2.6 (1.5)	2.7 (1.6)	2.2 (1.6)°
% medicine past month, prescribed	51	40	49	39
% medicine past month, non-prescribed	35	25	27	35
Fatigue past month^c^, mean (SD)	38.9 (20.3)	42.5 (24.2)	41.3 (23.2)	40.3 (20.5)
**OA–related symptoms**				
Duration of complaints, %				
< 1 year ago	4	2	3	3
1 to 3 years ago	21	26	23	24
3 to 10 years ago	35	34	34	41
10–20 years ago	23	17	21	18
> 20 years ago	17	21	18	15
Joints with complaints, %^b^				
0 joints	2	2	2	3
1 joint	24	22	25	16
2 joints	41	51	43	51
3 joints	20	18	18	22
4 joints	14	6	13	8
Pain severity past month^c^, mean (SD)	46 (24.1)	41 (25.0)	45 (24.2)	41 (24.5)
Pain tolerance past month^c^, mean (SD)	32 (21.2)	31 (22.8)	30 (21.7)	32 (20.7)
**Disability** (SIP-subscale physical functioning), mean (SD)	5.2 (5.4)	6.4 (8.5)	5.7 (7.6)	5.6 (6.0)
**Behavioral variables**, mean (SD)				
Attitude	37.8 (10.5)	38.1 (11.7)	36.2 (11.7)	40.7 (9.8)°°
Social norm	7.6 (4.0)	7.2 (4.3)	7.2 (3.9)	7.9 (4.4)
Self-efficacy	45.5 (26.0)	39.1 (22.9)	40.3 (24.1)	43.8 (25.3)
Intention, % yes	51	81**	58	77^a^
**Coping styles in general**, mean (SD)				
Active problem solving	2.2 (0.7)	2.4 (0.6)°	2.3 (0.7)	2.2 (0.5)
Seeking support	1.7 (0.5)	1.8 (0.6)	1.7 (0.5)	1.9 (0.6)*
Avoidance	2.2 (0.5)	2.2 (0.4)	2.2 (0.5)	2.1 (0.4)
Reacting religious	1.5 (0.7)	1.5 (0.7)	1.5 (0.7)	1.4 (0.6)
**Pain coping styles**, mean (SD)				
Pain transformation	2.2 (0.6)	2.0 (0.6)°	2.0 (0.6)	2.1 (0.6)
Distraction	2.6 (0.6)	2.2 (0.5)**	2.4 (0.6)	2.4 (0.6)
Reducing demands	2.2 (0.6)	2.3 (0.6)	2.2 (0.6)	2.4 (0.6)°°
Retreating	1.5 (0.5)	1.7 (0.5)°	1.6 (0.5)	1.7 (0.5)
Worrying about pain	1.7 (0.5)	1.8 (0.5)	1.7 (0.5)	1.8 (0.5)
Resting	2.1 (0.5)	2.4 (0.6)*	2.2 (0.6)	2.4 (0.6)°
**Participation intervention**, % yes	34	76**	45	68*
**Physical activity (Voorrips questionnaire)**, mean (SD)	13.0 (8.4)	10.6 (5.5)°°	12.5 (8.1)	10.6 (5.2)°

*°p* < 0.20; °°*p* < 0.10; *p < 0.05; **p < 0.01

^a^Significant difference between more vs. less and same activity behavior at 6 weeks on all marital status variables

^b^Significant difference between more vs. less and same activity behavior at 6 months on all levels

^c^Measured on a VAS scale, 0 = positive

### Predictors of Change in PA at 6 Weeks

The following variables were entered into the multivariate analysis based on associations with more PA behavior measured at 6 weeks: marital status, BMI, intention, general coping style “active problem solving,” pain coping styles, “pain transformation,” “distraction,” “retreating,” and “resting,” intervention status, baseline PA levels, and pain severity.

In the last step of the multivariate regression analysis (Table 4), intention, use of the pain coping style “resting,” and participation in the intervention were positively associated with more PA behavior whereas being single, having more severe pain, and making less use of the pain coping style “distraction” contributed to lower levels of PA behavior. Intention was the best predictor of change in PA behavior, followed closely by being single. The predictors explained 59% of the variance of more PA behavior. Demographic variables explained most of the variance together with intention and pain coping styles. No significant chi-square goodness-of-fit values were detected, and no multicollinearity was present. Missing analysis showed there were no significant differences between missing and valid subjects on demographic variables and BMI.

**Table 4:** Multivariate stepwise backward logistic regression of independent variables with 6 weeks change in activity behavior; *ß* value (*p* value), *n* = 70

Independent variables	Step 1	Step 2	Step 3	Step 4	Step 5
*1. Demographic variables* Marital status “single”^a^	−0.80 (0.28)	−0.85 (0.26)	−1.08 (0.18)	−2.06 (0.05)	−2.15 (0.05)
2. *Illness-related variables* Pain severity		−0.02 (0.09)	−0.02 (0.10)	−0.03 (0.06)	−0.03 (0.05)
3. *Behavior variables* Intention^b^			1.98 (0.00)	2.39 (0.00)	2.17 (0.01)
4. *Pain coping styles* Distraction Resting				−2.00 (0.01) 1.46 (0.03)	−1.81 (0.02) 1.50 (0.05)
5. *Confounder* Intervention^b^					1.31 (0.07)
**Total *R***^**2**^	**0.18**	**0.23**	**0.38**	**0.55**	**0.59**
**Incremental *R***^**2**^	**0.18**	**0.05**	**0.15**	**0.17**	**0.04**

^a^1 = married and living together

^b^1 = yes

### Predictors of Change in PA at 6 Months

Variables univariately associated with change in PA behavior at 6 months that were selected for multivariate analysis were sex, income, paid and voluntary work, age, BMI, comorbidity, attitude, intention, general coping style “seeking support,” pain coping styles “reducing demands” and “resting,” intervention status, and PA level at baseline. Again, pain severity was added.

Multivariate analysis showed that age was the only significant predictor (*n* = 64; *ß* =  −0.14; *p* = 0.02) of more PA behavior at 6 months and explained 40% of the variance (data not shown). In the model, higher age was associated with less PA behavior. Missing analysis showed that valid participants had higher income levels (*p* = 0.02) than those with missing data. No multicollinearity was present.

Self-reported PA levels (using the Voorrips questionnaire) categorized into high, medium, and low tertiles showed univariate associations with age, paid work activities, attitude, intention, general coping style “active problem solving,” baseline PA, and intervention status (data not shown). Stepwise backward logistic regression analysis showed age was the only significant predictor (*n* = 70; *ß* =  −0.13; *p* = 0.01). Again, higher age was associated with lower levels of PA behavior. The results from the two models are summarized in Table 5.

**Table 5:** Summary results, statistically significant associations between predictors and change in physical activity (PA)

	**At 6 weeks**	**At 6 months**
**Univariate**	Being single ↓	Age ↓
	Intention ↑	UCL seeking support ↑
	PCI distraction ↓	Intervention ↑
	PCI resting ↑	
	Intervention ↑^a^	
**Multivariate**	Being single ↓	Age ↓
	Pain severity ↓	
	Intention ↑	
	PCI resting ↑	
	PCI distraction ↓	
	Intervention^a^ ↑	

*PCI* pain coping inventory, *UCL* Utrecht coping list

↑ positive association indicating higher value of predictor leads to more PA

↓ negative association indicating higher value of predictor leads to less or same level of PA

^a^Confounder

## Discussion

This study explored predictors of self-reported change in PA among older adults with radiologic and clinical confirmed knee or hip OA. Six weeks after baseline, the predictors accounted for 59% of the variance in the outcome variable, and after 6 months, this was 40%. Compared with baseline, almost half of the participants indicated that they had increased their PA behavior at 6 weeks, and by 6 months, this was 37%. This finding is consistent with other studies, as the effect of change in behavior is difficult to maintain over time [31]. Even so, only a small percentage (10%) indicated that they had reduced their PA.

Of the behavioral aspect of the ASE model, only the intention to become more physically active item predicted becoming more physically active at 6 weeks, although the variables influencing intention, i.e., attitude, self-efficacy, and social norm, were not associated. This result is contrasted to other studies which show that self-efficacy in particular plays a role in PA [18, 32]. This could be due to the relatively small groups in our study and the fact that the intervention and control groups were analyzed together. Self-efficacy was in fact one of the significant results in the RCT [21], and we treated the intervention as a confounder in the analyses.

Although an intention-behavior gap does exist, intention, however, is still known as one of the best predictors of PA [33].

Results on the pain coping styles “resting” and “distraction” seem to contradict other studies that used the same instrument (PCI), where active pain coping styles such as distraction were found to be cross-sectionally associated with more sporting activity, and passive coping styles such as resting with less sporting activity [15]. The use of the pain coping style “resting” also has been shown to be a predictor of future limitations in patients with knee and hip OA [6]. These studies used OA patients recruited through rehabilitation centers which might mean more pain and subsequent limitations in their study sample. Patients might use different coping strategies when faced with more serious symptoms.

Given the fact that pain severity also predicted PA at 6 months, the association with pain coping becomes more relevant. The interaction between these aspects makes the relationship between OA and PA complex. On the one hand strenuous occupational tasks and high-intensity competitive sports are risk factors for OA of the hand, hip, and knee [34, 35], while longitudinal studies in older adults with knee or hip osteoarthritis show that higher PA levels have been associated with a slower decline in function [5]. Examining how coping mechanisms play a role and using this information might help individuals to gain better control of the disease symptoms that influence PA and the pathway to disability [36].

In our sample, being single and having more severe pain were also associated with less PA behavior at 6 weeks. Both factors are well known to negatively influence PA behavior [13, 14, 30].

At 6 months, the behavioral aspects showed only univariate associations with self-reported behavioral change and PA levels. When controlled for other demographic and health-related predictors, no relationship with behavioral aspects was found. Only higher age predicted less PA at 6 months. This outcome confirms other studies that have reported higher age being associated with less PA behavior [13]. Nevertheless, it is striking that only one predictor remained to explain change in PA behavior as well as self-reported PA levels. From univariate results, it can be seen that the more physically active group is on average 3 years younger, which is quite a large gap in an age range of 20 years. In this group also, twice as many participants were active in paid or voluntary work which contributes to higher baseline PA levels [37] which could make it difficult to further increase PA levels in this group. Alternatively, age may stand for other variables that change with age but were not included in our set of predictors, or were not sufficiently specified, i.e., the variable for chronic disease which did not specify type of disease.

Other well-known predictors of change in PA identified in the literature such as BMI, number of chronic diseases, and disability [38, 39] were in our study not significantly related to self-reported change in activity behavior. Differences between study populations could explain this discrepancy. The high variance explained by our multivariate model might indicate that enough important variables were included for our study population. Because peer education was an important part of the intervention (see Textbox 1), it is plausible that all predictors of intention (attitude, self-efficacy, and social norms in the ASE model) were positively influenced leading to a strong intention for changing behavior in PA. Indeed, we found in the earlier RCT [21] a moderate effect of the intervention on self-efficacy.

### Limitations of This Study

Certain limitations of this study should be considered. An important limitation of this study was the small study population, which decreased the statistical power meaning that long-term multivariate associations of self-reported change in PA behavior could not be assessed. Even so, no multicollinearity was present and the models showed good fit. Identified univariate predictors should give a good indication of the variables to be used in other studies.

Another limitation is self-reported behavior, which may be unreliable in assessing change in behavior. Socially desirable answers could be given. In addition, levels of PA tend to be overestimated [40]. Comparison with PA levels as measured with a validated questionnaire [22] in our study showed that subjective changes corresponded quite well with reported PA levels. Also, similar results were shown for predictors of 6 months PA between the self-reported change and levels outcome.

Despite these limitations, univariate and multivariate findings of this study should be considered or taken into account for future interventions. Higher age in particular was shown to be a high risk for lower PA behavior. Similar to other studies, the ASE model and others can be recommended to provide a background for interventions [41, 42], or at least behavior-guided interventions [31, 43].

It can be safely stated that these and other results show that the relationship between OA and PA is complicated and needs further study [44–46]. The interplay in time between the disease and its symptoms, functional limitations, and psychological reactions such as coping styles is complex and if left unattended may lead to a downward spiral of avoidance, fear of activity, and subsequent deterioration in function and disability [47, 48].

## Conclusion

In conclusion, a change in PA behavior in the short term was found to be related to behavioral factors especially intention and also pain coping styles. This, together with the other predictors of self-reported change, in particular pain severity and marital status, should be addressed in designing more effective future interventions. The intervention that we used could be a good starting point for reaching that goal.

## Supplementary Information

Below is the link to the electronic supplementary material.

Supplementary file1 (DOCX 22 KB)

## Footnotes

1 Petty BJ, Cusack SA. Assessing the impact of a seniors' peer counseling program. Educ Gerontol 1998;15:49–64.

2 In Dutch, the word *lichaamsbeweging* was used. This includes besides exercising also other physical activities such as walking, biking, doing household activities, etc.
