# Supplementary material for: What Are the Predictors of Self-Reported Change in Physical Activity in Older Adults with Knee or Hip Osteoarthritis?
Source: Int J Behav Med. 2022 Mar 23;30(2):199–210. doi: 10.1007/s12529-022-10080-y (PMC10036423; doi:10.1007/s12529-022-10080-y)
Supplement: Supplementary file 1 — Supplementary file1 (DOCX 22 KB) [file 12529_2022_10080_MOESM1_ESM.docx]

**CALL PROTOCOL FOR TELEPHONE MOTIVATIONAL INTERVIEWING 1(Physical Activity):**

**Introduction:**

☺ **Good evening/afternoon/morning. You are speaking with** __________ (interviewer name). **I would like to speak to** _______ (Mr. or Mrs. + full name).

☺ **You speak with** _______ (name of interviewer), **the HEALTH COACH of the Vitalum study of Maastricht University. Am I speaking with** _________ (Mr. or Mrs. + full name) **born on** _________ (date of birth)?

If yes -> continue

If no ->: ask for the right person or set up a time for a follow-up call

☺ **As you may recall, you have registered to participate in the Vitalum study of Maastricht University. As part of this research, we conduct conversations with people about physical activity and nutrition. You have been selected to participate in Vitalum. This means that we will call you 4 times in the coming year. In these phone calls we will talk about your diet or exercise activities. The phone call will take approximately 20-30 minutes. Do you currently have the time and opportunity for the conversation?**

If yes -> continue with the script

If no -> make a new appointment (date + time) and close the conversation. Note the date + time of the appointment.

☺ **We would like to let you know that this conversation is being recorded so that its quality can be checked. All information revealed in this conversation is confidential and will be treated anonymously.**

☺ **What topic would you most like to talk about today: fruit / vegetable intake or physical activity?**

If physical; activity -> continue with this script + NOTE

If nutrition -> continue with the script of vegetable / fruit + NOTE

**Estimation of current behavior:**

☺ **In the questionnaire you completed some time ago, you indicated that you are physically active with a moderate-intensity for approximatel**y ________ (number of hours of moderate intensity per week) **hours per week and that you get** ________ **days** (number of days per week) **at least** **30 minutes of moderate physical activity.**

☺ **You also indicated that you are physically active in the following ways**: ___________ (name types of exercise, e.g. housework, walking to work, etc.). **Do you have anything to add to this?**

If no -> move on to the next question

If yes -> NOTE Use basic techniques

☺ **To what extent has your physical activity pattern changed in the past month?** NOTE. Use basic techniques. **OR** question: **Have you continued to exercise more, less or at the same level in the past month?**

If more/more intensively -> ☺ **You indicate that you have started exercising** _____ (more / more intensively) **in the past month**. **That is amazing! Would you like to tell me more about that?** NOTE. Use basic techniques.

CONTINUE WITH NORM

If less (intensive)-> ☺ **Would you like to tell me why you started exercising less (or less intensively)?** NOTE. Use basic techniques.

If no change -> Continue with norm

**NORM:**

☺ **If it is okay with you, I would like to talk a little bit about how much physical activity you think is minimally needed for good health (according to the Dutch Standard for Healthy Exercise)**?

If yes -> Have participant tell ‘norm’ and correct if necessary (see MOVEMENT CARD for additional information if necessary) using the following question:

If no -> Continue to next question.

☺ **Would you like to know how much you should minimally exercise according to these Dutch guidelines?**

If no -> Continue with importance / confidence

If yes -> ☺ **According to the Dutch guidelines, it is important that you exercise moderately/medium-intensity for at least 30 minutes a day at least 5 days a week. Moderately or moderately intensive means…**

Ages 45 to 54 -> … **that you, for example, walk at least 5 km/h or cycle 16 km/h.**

55 to 70 years old -> … t**hat you, for example, walk at least 4 km/h or cycle 10-12 km/h.**

MORE INFORMATION ABOUT EXERCISE & HEALTH SEE MOTION CARD

☺ **What do you think about this?** NOTE. Use basic techniques.

Try to **summarize** the **current situation** regarding physical activity.

**Importance + confidence:**

☺ **If it's okay with you, I'd like to ask you some questions about your motivation and confidence to start exercising more.**

If no -> ☺ **You indicate that you do not want to talk about this. Can you tell me why not?** Use basic techniques.

If yes -> **Proceed to estimate importance.**

**Estimate IMPORTANCE:**

☺ **Can you indicate on a scale of 0-10 how important it is to you to exercise more? 0 means that you do not find it important to exercise more at all and 10 that you find it very important to exercise more.** NOTE.

Why not LOWER?

If answer is 0-1 -> ☺ **For importance, you chose a ___ (state number). Tell me why you chose a ___ (state number)?** NOTE. Use basic techniques.

If answer is 2-10 -> ☺ **For importance, you chose a ___ (state number). Tell me why you chose a** ___ (state a number) **instead of a** ___ (name 3 to 4 numbers lower, for example someone gave an 8 and now you ask why an 8 instead of a 5)? NOTE. Use basic techniques.

If the respondent has difficulty answering this question, you can also ask why the respondent does think it is important to move more (benefits more exercise). Then use basic techniques.

Continuation estimate importance:

Why not HIGHER?

The answer is 0-4 -> ☺ **Why did you chose a** ___ (state a number) **instead of a 7 or 8?** NOTE. Use basic techniques.

The answer is 5-8 -> ☺ **Why did you chose a** ___ (state a number) **instead of a 9 or 10?** NOTE. Use basic techniques.

If the respondent has difficulty answering this question, you can also ask why the respondent thinks it is less important to exercise more (disadvantages of exercising more). Then use basic techniques again.

☺ **What needs to be done to move to a higher grade?**

If answer is 0-4 -> ☺ **What can you do to increase the importance of exercising more to a 7 or 8?** NOTE. Use basic techniques.

If answer is 5-9 -> ☺ **What can you do to increase the importance of exercising more to a 9 or 10?** NOTE. Use basic techniques.

The answer is 10 -> Confirm the importance with a compliment and move to trust.

If people have trouble answering these questions of the importance ruler, first choose the **values ​​clarification**, see CHANGE TALK card.

Try to **summarize** the **importance** of getting more exercise.

**Estimate Confidence:**

If a person finds it absolutely not important to exercise more (0 or 1), ask him/her the confidence question in hypothetical form: ☺ **Suppose you think it is very important to exercise more, how much confidence do you have in it**… (see below).

☺ **On a scale of 0 to 10, how confident are you that you could manage to exercise more? Here 0 means that you have no confidence at all and 10 that you have a lot of confidence to start exercising more.** NOTE

*Confidence refers to the respondent's ability to move in any situation.

Why not LOWER?

The answer is 0-1 -> ☺ **For confidence, you chose give a** ___ (state a number). **Tell me why you chose a** ___ (state number)? NOTE. Use basic techniques.

The answer is 2-10 -> ☺ **For confidence, give a** ___ (state a number). **Tell me why you chose a** ___ (state a number) **instead of a** ___ (name 3 to 4 numbers lower, for example someone gave an 8 and now you ask why an 8 instead of a 5)? NOTE. Use basic techniques.

If the respondent has difficulty answering this question, you can also ask if he/she can explain why he/she is confident that he/she can exercise more. Use basic techniques.

Continued estimate Confidence:

Why not HIGHER?

The answer is 0-4 -> ☺ **Tell me why you chose a** ___ (state a number) **instead of a 7 or 8?** NOTE. Use basic techniques.

The answer is 5-8 -> ☺ **Tell me why you chose a** ___ (state a number) **instead of a 9 or 10?** NOTE. Use basic techniques.

If the respondent has difficulty answering this question, you can also ask if he/she can explain why he/she has less confidence that he/she can move more. Use basic techniques.

☺ **What needs to be done to move to a higher grade**?

If answer is 0-4-> ☺ **What can you do to increase your confidence level to a 7 or 8?** NOTE. Use basic techniques.

If answer is 5-9 -> ☺ **What can you do to increase your confidence level to a 9 or 10?** NOTE. Use basic techniques.

The answer is 10 -> Confirm trust with a compliment and proceed with action plan/agreements.

If the respondent has difficulty answering the 'what should be done' question, you can also ask in which ways he/she could move more. NOTE. Use basic techniques.

If a respondent has difficulty with the confidence ruler questions, see the CHANGE TALK card.

Try to **summarize** the **confidence** to exercise more.

**Action plan/agreements:**

☺ **To what extent do you plan to exercise more in the coming weeks?** NOTE. Use basic techniques.

If the person **does not intend** to move anymore ->

☺ T**ell me why you do not intend to exercise more?** NOTE. Use basic techniques, (examine lack of interest/ambivalence (advantages/disadvantages) through open-ended questions. Listen to the respondent and respect his/her decisions.

☺ **To what extent are you willing to think about getting more exercise in the coming weeks?** / **Because exercise is very healthy physically and mentally, I would like to encourage you to think about more exercise.** NOTE Continue with closing

If the person **does intend** to exercise more ->

☺ **How good that you want to move more! What would you like to do about your amount of exercise in the coming weeks?** NOTE. Use basic techniques (setting goals).

☺ **In what ways do you think you could exercise** ___________ (state purpose)? NOTE. Use basic techniques (brainstorming about possible changes).

☺ **Which of these ways to move more would you like to try in the near future?** NOTE.

☺ **What things could prevent you from getting physically active and how do you go about this**? NOTE. Use basic techniques (support respondent in dealing with barriers/obstacles and suggest solutions if necessary).

Try to **summarize t**he **pledge** and **action plan.** Continue with closing.

**Follow-up action plan/appointments:**

When the person is in the preparation/action/ phase (the person is starting/has started to move according to the norm->

☺ I**t's great that you started moving more! Tell me how you managed to exercise more?** NOTE. Use basic techniques (giving positive feedback and encouraging person to continue with behavior, asking open-ended questions about how the respondent managed to change).

☺ **To what extent do you encounter things that make it difficult for you to keep moving?** NOTE. Use basic techniques (deepening barriers and concerns and brainstorming solutions from others).

☺ **To what extent do you intend to exercise at the same level or more?** NOTE. Use basic techniques (promise to stay on the same level or to move more).

If the person **does not intend** to exercise at the same level/more-> Examine lack of interest through open questions. Listen to the respondent and respect his/her decisions. Continue with closing.

If the person **does intend** to move more-> Confirm and continue with closing.

**Closure:**

☺ **If it's okay with you, I'd like to summarize what we discussed in this phone call**. If consent is given, proceed.

Start repeating the **cons (**negative reasons) to exercise more.

Then list the benefits (**positive r**easons) for exercising more.

Also state the goal the respondent wants to achieve or **agreements** that have been made.

☺ **Would you like to add or comment on what I just said?** NOTE. Use basic techniques.

☺ **Do you have any comments or questions?** NOTE.

☺ **The next phone call will take place in about 2 months, so that will be in** ________________ (name month).

☺ **Thank you very much** ________ (respondent's full name) **for your time and commitment. Have a good morning/afternoon/evening.**
